# Changes in Metabolism and Mitochondrial Bioenergetics during Polyethylene-Induced Osteoclastogenesis

**DOI:** 10.3390/ijms23158331

**Published:** 2022-07-28

**Authors:** Nur Shukriyah Mohamad Hazir, Nor Hamdan Mohamad Yahaya, Muhamad Syahrul Fitri Zawawi, Hanafi Ahmad Damanhuri, Norazlina Mohamed, Ekram Alias

**Affiliations:** 1 Department of Biochemistry, Faculty of Medicine, Pusat Perubatan Universiti Kebangsaan Malaysia, Jalan Yaacob Latif, Bandar Tun Razak, Kuala Lumpur 56000, Malaysia; nurshukriyah@unikl.edu.my (N.S.M.H.); hanafi.damanhuri@ppukm.ukm.edu.my (H.A.D.); 2 Clinical Laboratory Section, Institute of Medical Science Technology, Universiti Kuala Lumpur, A1-1, Jalan TKS 1, Taman Kajang Sentral, Kajang 43000, Selangor, Malaysia; 3 Department of Orthopaedics, Faculty of Medicine, Pusat Perubatan Universiti Kebangsaan Malaysia, Jalan Yaacob Latif, Bandar Tun Razak, Kuala Lumpur 56000, Malaysia; nhmycj@gmail.com; 4 Department of Orthopaedics, School of Medical Sciences, Universiti Sains Malaysia, Kubang Kerian, Kota Bharu 16150, Kelantan, Malaysia; msf.zawawi@usm.my; 5 Department of Pharmacology, Faculty of Medicine, Pusat Perubatan Universiti Kebangsaan Malaysia, Jalan Yaacob Latif, Bandar Tun Razak, Kuala Lumpur 56000, Malaysia; azlina@ppukm.ukm.edu.my

**Keywords:** polyethylene, polyethylene-induced osteolysis, osteoclast, osteoclastogenesis, reactive oxygen species, mitochondrial bioenergetics, substrate oxidation, glycolysis, vitamin E, tocotrienol

## Abstract

Changes in mitochondrial bioenergetics are believed to take place during osteoclastogenesis. This study aims to assess changes in mitochondrial bioenergetics and reactive oxygen species (ROS) levels during polyethylene (PE)-induced osteoclastogenesis in vitro. For this purpose, RAW264.7 cells were cultured for nine days and allowed to differentiate into osteoclasts in the presence of PE and RANKL. The total TRAP-positive cells, resorption activity, expression of osteoclast marker genes, ROS level, mitochondrial bioenergetics, glycolysis, and substrate utilization were measured. The effect of tocotrienols-rich fraction (TRF) treatment (50 ng/mL) on those parameters during PE-induced osteoclastogenesis was also studied. During PE-induced osteoclastogenesis, as depicted by an increase in TRAP-positive cells and gene expression of osteoclast-related markers, higher proton leak, higher extracellular acidification rate (ECAR), as well as higher levels of ROS and NADPH oxidases (NOXs) were observed in the differentiated cells. The oxidation level of some substrates in the differentiated group was higher than in other groups. TRF treatment significantly reduced the number of TRAP-positive osteoclasts, bone resorption activity, and ROS levels, as well as modulating the gene expression of antioxidant-related genes and mitochondrial function. In conclusion, changes in mitochondrial bioenergetics and substrate utilization were observed during PE-induced osteoclastogenesis, while TRF treatment modulated these changes.

## 1. Introduction

Bone is an important organ that serves multiple functions, including muscular attachment and body movement, blood cell production, and protection of vital organs. The structural integrity of healthy bone results from a good balance between bone resorption (by osteoclasts) and new bone formation (by osteoblasts) [1].

Differentiation into mature and functional osteoclasts starts with the binding of a receptor activator of nuclear factor kappa-B ligand (RANKL) to RANK [2] at the membrane of pre-osteoclasts. The expression of RANK can be stimulated by macrophage colony-stimulating factor (MCSF) [3]. Several pathways are stimulated following the binding of RANKL to RANK [4,5]. These pathways further induce the expression of NFATc1, the key transcription factor that regulates and drives the osteoclast differentiation program [6]. The importance of the NFATc family in inducing differentiation into multinucleated osteoclasts has been studied by Ikeda et al. (2004), where treatment with RANKL was observed to induce nuclear localization of NFATc1 [7].

Polyethylene (PE) is a material commonly used as a lining to reduce friction against implant bearing surfaces. Metal prostheses with polyethylene lining are the most popular choice in joint replacement surgery. Continuous friction of the polyethylene lining over a long period of use will generate PE particles, which trigger an inflammatory response in surrounding tissues [8,9]. Both crosslinked and non-crosslinked types of PE induce an inflammatory response in tissues, with the earlier one producing a stronger response [10]. These PE particles are believed to be engulfed by macrophages, causing them to release cytokines such as interleukin-1 and tumor necrosis factor, leading to inflammation within the surrounding tissues [11]. The literature has demonstrated elevated RANKL in tissues of peri-implant osteolysis [12,13]; therefore, it is widely accepted that the increase in RANKL is representative of peri-implant osteolysis [8]. The release of RANKL also causes the activation of tissue macrophages to be recruited to the site of inflammation. These tissue macrophages differentiate into osteoclasts, which are capable of resorbing bones that hold the implant, causing the implant to loosen [14]. PE has been shown to stimulate the formation of osteoclasts and increase their resorption activity both in vitro [15] and in vivo [16].

Osteoclasts are highly energy-consuming cells [17]. The function of osteoclasts in degrading bone requires a large amount of energy, which is needed to pump out protons and degrading enzymes. Adenosine triphosphate (ATP), a currency of chemical energy, is mainly produced by mitochondria. A recent study has found that most ATP is produced through oxidative phosphorylation (OXPHOS) [18]. Studies have shown that differentiated human osteoclasts, as well as osteoclasts from rats and mice, possess high numbers of mitochondria within each cell [19,20,21,22]. It has also been observed that the mitochondria in mature osteoclasts have higher levels of enzymes needed for the tricarboxylic acid cycle (TCA) and oxidative phosphorylation, compared to immature ones [17]. Previous studies have demonstrated that osteoclastogenesis in bone-marrow macrophages (BMMs) could be disturbed through the inhibition of mitochondrial complexes [23,24]. Studies have also found that, during RANKL-induced osteoclastogenesis, mitochondrial biogenesis is induced through PGC-1β and alternative NF-Kβ signaling [25,26].

Findings from previous studies suggest that changes in cellular metabolism also take place during osteoclastogenesis. Kim et al. (2007) observed increases in glucose metabolism, oxygen consumption, and lactate production during RANKL-induced osteoclastogenesis [23]. A later study observed an increase in the expression of glucose transporter 1 (Glut1), as well as increased expression of glycolytic genes, during osteoclastogenesis [27]. A recent study shows that both progenitor and mature osteoclasts utilize glucose as the main energy source, compared to glutamine and fatty acids [28]. Lactate dehydrogenase activity has also been found to be high during RANKL-induced osteoclast differentiation, suggesting the utilization of lactate and increased extracellular acidification [29]. Taken together, these findings suggest that increases in cellular metabolism and glycolysis may take place during RANKL-induced osteoclastogenesis. However, in the context of PE-induced osteoclastogenesis, the changes in cellular metabolism during the process have not previously been studied.

In general, reactive oxygen species (ROS) produced in macrophages play a vital role in cellular defense. Their accumulation could promote RANKL-induced osteoclastogenesis [30,31,32]. The accumulation of ROS (e.g., superoxide anion) and ROS-producing enzymes could stimulate osteoclast formation and increase bone resorption [33,34,35,36]. NADPH oxidases are the main enzymes that promote the production of ROS in osteoclasts [37,38]. Previous studies have demonstrated that PE particles resulted in the accumulation of the oxidative stress marker malonylaldehyde (MDA) in the blood of rats [39]. OXPHOS, which provides energy to the cell, is another source of ROS, as ROS are released through complex II [40].

Tocotrienols are a subclass of the vitamin E family comprised of four isomers: α-tocotrienol, β-tocotrienol, γ-tocotrienol and δ-tocotrienol [41]. Tocotrienols-rich fraction (TRF) refers to a combination of naturally available α-tocopherol and tocotrienols (comprising more than 50% of the total fraction) [42]. Tocotrienols have been widely studied for their antioxidant properties in bone and in other diseases [43,44,45,46]. Our previous work has shown that tocotrienols are beneficial for bone health because they can promote bone regeneration both in vitro [47,48] and in vivo [49,50,51]. In a systematic review by Radzi et al. (2018), it was concluded that TRF is able to decrease resorption activity, but not osteoclastogenesis [52]. The effect of TRF in exhibiting protective properties in the context of PE-induced osteolysis has never been studied. It is also interesting to explore whether the modulation of PE-induced osteolysis by TRF treatment has an impact on the mitochondria in osteoclasts.

In this study, we focus on the changes in mitochondrial bioenergetic parameters, glycolysis efficiency, and profile of substrates fueling mitochondrial respiration during PE-induced osteoclast differentiation, and how these could be affected by TRF treatment. From our published systematic review [53]—even though a majority of the literature has reported an increase in osteoclastogenesis following hypoxia—some studies have indicated otherwise. This may suggest that both aerobic and anaerobic respiration are important during osteoclastogenesis; hence, both OXPHOS and glycolysis, as well as the oxidation of metabolic substrates, should be examined in osteoclasts.

## 2. Results

### 2.1. Confirmation of PE-Induced Osteoclast Differentiation and Activity

**Figure 1:**
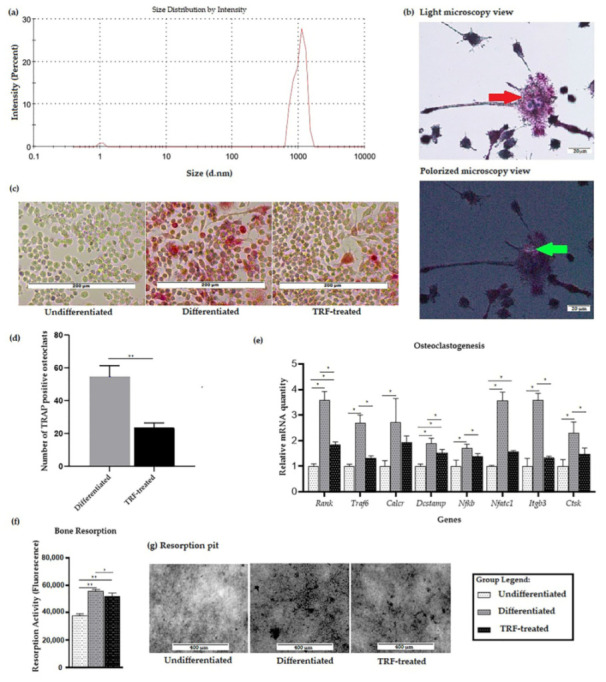
Validation of PE-induced osteoclastogenesis in vitro: (**a**) Distribution of particle size of PE particles. (**b**) Micrograph showing multinucleated cells (red arrow) formed after exposure to PE particles following RANKL induction. PE particles (bi-refringence under the polarized view, green arrows) were seen in close proximity to macrophages/osteoclasts stained with hematoxylin and TRAP (200× magnification). (**c**) Micrograph showing TRAP staining of three different groups (100× magnification). (**d**) TRAP-positive osteoclasts were counted over a 208 mm^2^ area using 100× magnification. Average of five areas per well for differentiated group and TRF-treated group were subjected to *t*-test analysis (**e**) Expression profiles of osteoclast-related genes. Data are presented as relative mRNA quantity ± SD, normalized to the undifferentiated group. (**f**) The resorption activity of three different groups was measured as fluorescence activity. (**g**) Resorption pit formation (represented from the black dots) in three different groups. ANOVA followed by Tukey’s post-hoc analysis was performed for data in (**d**–**f**). * indicates *p* < 0.05 and ** indicates *p* < 0.01.

We first performed particle size analysis on the polyethylene particles used throughout this study. The z-average of the sample was 2132 (nm) in diameter, with a polydispersity index (PdI) of 0.372. The minimum particle size, based on peak one, was 1.047 nm ± 0.07 (diameter), while the maximum particle size, based on peak two, was 1051 nm ± 0.2047 (diameter); see Figure 1a.

Cells exposed to polyethylene particles and RANKL differentiated into multinucleated osteoclast-like cells. The formation of osteoclast-like cells was confirmed using a light microscope, as shown in Figure 1b. Polyethylene particles were seen as bi-refringence under a polarized filter. After day 9, cells were stained with TRAP and nuclei were counterstained with Gill’s hematoxylin. No TRAP-stained cell was observed in the undifferentiated RAW264.7 cells. A higher number of TRAP-positive cells and more intense staining was observed in the differentiated group, compared to the undifferentiated group (Figure 1c). Meanwhile, in the TRF-treated group, the staining intensity was less than in the differentiated group. The TRF-treated group also showed a significantly lower number of TRAP-positive osteoclasts, compared to the differentiated group (*p* < 0.05).

A significantly higher expression of the osteoclast-associated genes *Rank*, *Dcstamp*, *Nfatc1*, *Traf6*, *Calcr*, *Nfkb*, *Itgb3*, and *Ctsk* was observed in the differentiated cells compared to the undifferentiated ones. Higher expression of *Rank*, *Dcstamp*, and *Nfatc1* was also observed in the TRF-treated cells compared to the undifferentiated cells. Lower expression of *Rank*, *Traf6*, *Dcstamp*, *Nfatc1*, *Itgb3*, and *Ctsk* genes was observed in the TRF-treated cells relative to the untreated differentiated cells (Figure 1d).

In the bone resorption assay, the osteoclast activity—represented by the total area of resorption on the synthetic carbonate appetite (CaP), which mimics the dentin disc—was measured from the fluorescence signal. Statistically higher bone resorption was observed in both differentiated and TRF-treated cells compared to the undifferentiated cells. The TRF-treated cells had a significant lower resorption activity compared to the differentiated ones (*p* < 0.05; Figure 1e).

### 2.2. ROS Production

**Figure 2:**
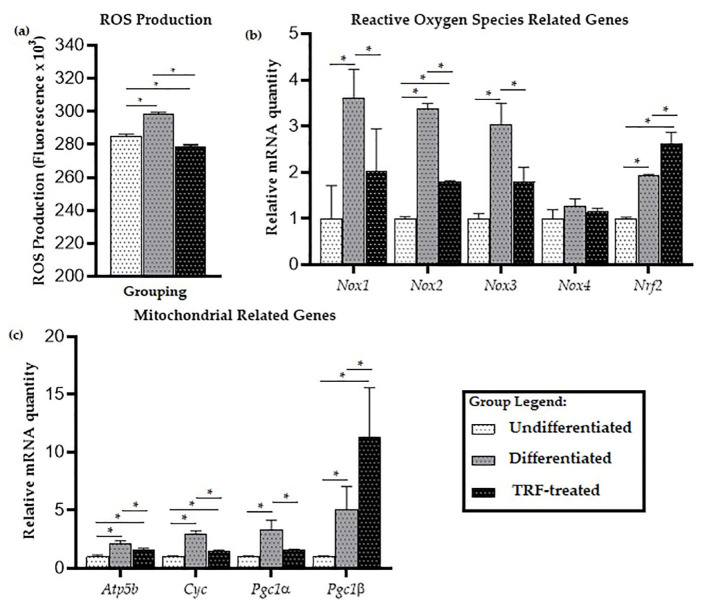
ROS production and expression of mitochondria-related genes: (**a**) The levels of ROS, (**b**) expression of oxidative stress-related genes, and (**c**) mitochondria-related genes in undifferentiated, untreated, and TRF-treated differentiated cells. ANOVA and Tukey’s post-hoc analysis were performed (**a**–**c**). * indicates *p* < 0.05. Data are presented as relative quantity ± SD, normalized to the undifferentiated group.

When the cells were exposed to PE particles, it was observed that the differentiated cells produced higher ROS levels (*p* < 0.05) than undifferentiated cells. TRF treatment of the differentiated cells significantly lowered the ROS level (Figure 2a).

For oxidative stress-related genes, there was significantly higher expression of genes encoding NADPH oxidase enzymes *Nox1*, *Nox2,* and *Nox3* in the differentiated cells, compared to the undifferentiated ones (*p* < 0.05), while treatment with TRF reduced the expression of these genes (*p* < 0.05). There was no significant difference in the expression of *Nox4* between all groups (Figure 2b). Higher expression of the antioxidant-related gene *Nrf2* was observed compared to undifferentiated cells (*p* < 0.05). Relative to the undifferentiated group, *Nrf2* expression was higher in the differentiated group, and even higher in the TRF-treated group (*p* < 0.05).

### 2.3. Mitochondrial-Related Genes Expression

Significantly higher expression of mitochondria-related genes *Atp5b*, *Cyc*, *Pgc1a,* and *Pgc1b* was observed in the differentiated cells compared to the undifferentiated cells; while lower expression of *Atp5b*, *Cyc*, and *Pgc1a* was observed in the TRF-treated cells compared to the differentiated cells (*p* < 0.05). Higher mRNA expression of Pgc1b was seen in the TRF-treated differentiated cells compared to the untreated ones (*p* < 0.05; Figure 2c).

### 2.4. Cellular Metabolic Changes

The higher expression of mitochondria-related genes observed in the differentiated cells prompted us to investigate the changes in mitochondrial bioenergetic activity during osteoclast differentiation.

**Figure 3:**
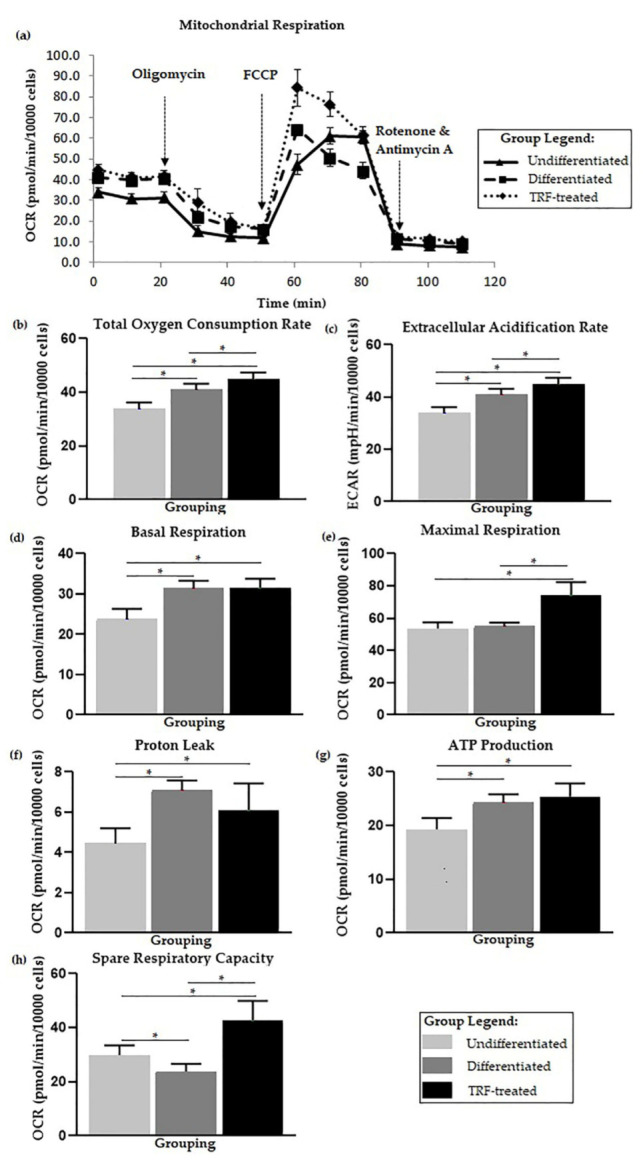
Cellular metabolic changes. Higher basal respiration in differentiated cells and treatment with TRF improves mitochondrial bioenergetic function. (**a**) Overall flux analysis graph, which is further analyzed from multiple aspects of mitochondrial bioenergetic functions: (**b**) Total oxygen consumption rate, (**c**) Extracellular acidification rate, (**d**) Basal respiration, (**e**) Maximal respiration, (**f**) Proton leak, (**g**) ATP production, and (**h**) Spare respiratory capacity. Note: Bioenergetic parameters (**d**–**h**) were calculated from each event. Parameters (**b**–**e**) were analyzed using one-way ANOVA followed by Tukey’s multiple comparison test, while (**f**–**h**) were analyzed using Welch ANOVA followed by the Games–Howell multiple comparison test. * indicates *p* < 0.05.

The kinetic profile of the oxygen consumption rate is displayed in Figure 3a. The highest total OCR and ECAR were observed in the TRF-treated group, followed by the differentiated and undifferentiated groups, and the mean significantly differed (*p* < 0.05) between groups (Figure 3b,c).

Higher basal cell metabolism, proton leak, and ATP production were observed in the differentiated group (*p* < 0.05) compared to the undifferentiated one. No statistical difference was observed for maximal respiration between undifferentiated and differentiated cells. Lower spare respiratory capacity was observed in the differentiated cells (*p* < 0.05) than the undifferentiated cells (Figure 3d–h).

The highest maximal respiration was observed in the TRF-treated group (*p* < 0.05) when compared to the undifferentiated and untreated differentiated groups. ATP production was also higher in the TRF-treated group (*p* < 0.05) compared to the undifferentiated and untreated differentiated groups. The TRF-treated cells also showed lower proton leak activity (*p* < 0.05) than in the untreated differentiated cells. The TRF-treated cells also exhibited higher spare respiratory capacity (*p* < 0.05) compared to the differentiated group and undifferentiated cells (Figure 3d–h).

### 2.5. Glycolysis Stress Assay

**Figure 4:**
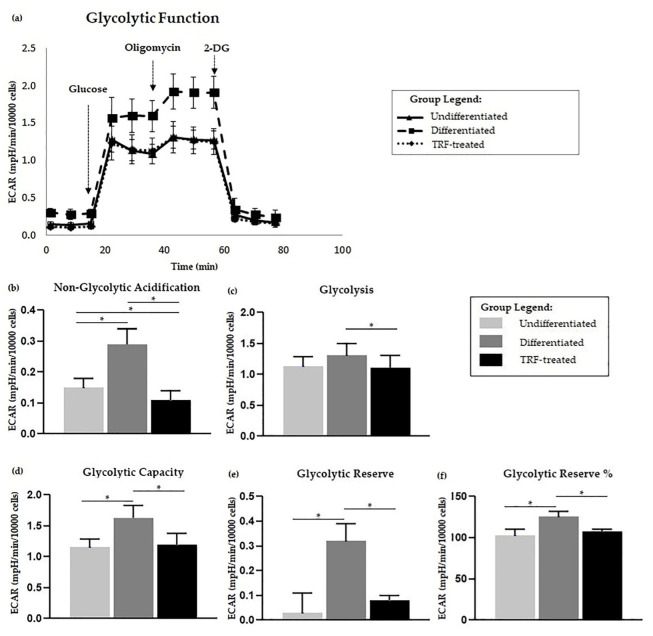
Glycolysis stress assay. Polyethylene-induced differentiation and TRF upon glycolysis stress assay. (**a**) Extracellular acidification rate (ECAR) of overall flux analysis normalized to 10,000 cells. Comparisons between groups in glycolysis stress test parameters; (**b**) non-glycolytic acidification, (**c**) glycolysis, (**d**) glycolytic capacity, as well as (**e**) glycolytic reserve and (**f**) percentage, are shown above. All measured parameters in (**b**–**f**) were analyzed using one-way ANOVA followed by Tukey’s multiple comparison test. * indicates *p* < 0.05.

The kinetic plot showing ECAR following each event is displayed in Figure 4a. Based on the ECAR readings, there was no significant difference in glycolysis activity in differentiated cells compared to the undifferentiated ones (Figure 4c). However, the differentiated cells were higher in other measured parameters, including non-glycolytic acidification, glycolytic capacity, glycolytic reserve, and glycolytic reserve percentage (Figure 4b,d–f) relative to the undifferentiated cells. Treatment with TRF resulted in lower glycolysis and all other glycolysis stress test parameters compared to the differentiated group (*p* < 0.05).

### 2.6. Mitochondria Substrate Oxidation Profile

Considering the changes in mitochondrial activity presented above, we were interested in assessing the changes in the substrates that fuel the mitochondrial bioenergetic activity during PE-induced osteoclastogenesis and how TRF treatment may modulate these changes.

**Figure 5:**
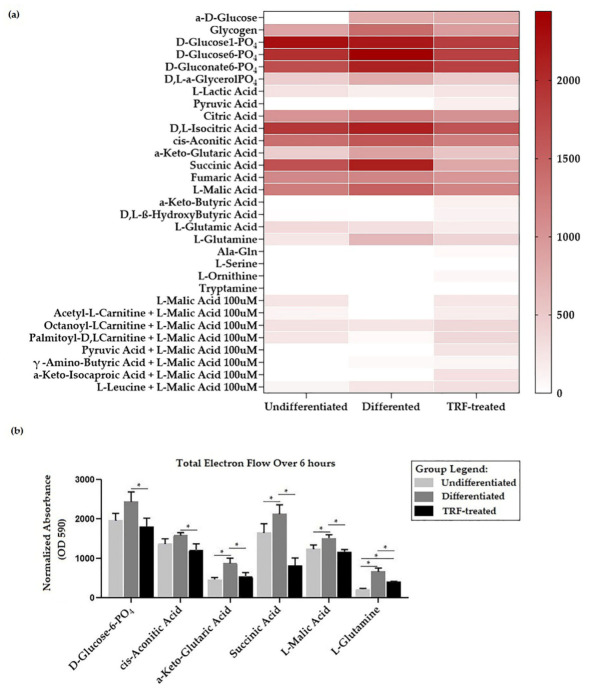
Mitochondria substrate oxidation profile. (**a**) Heatmap showing the rate of electron flow for 31 substrates. (**b**) Graph of total electron flow of substrates, indicating significant differences between groups. * indicates *p* < 0.05. Note: Total electron flow over time was determined by calculation of the area under the curve for absorbance reading taken over 6 h of kinetic measurements.

The heatmap in Figure 5a shows that most of these substrates oxidized at different rates, where a higher total electron flow is represented by a darker shade of red and no oxidation is represented by white. Significant differences between groups were observed in several substrates, as presented in Figure 5b. Total electron flow over six hours under different conditions were found to differ statistically for six substrates: D-glucose-6-phosphate [F(2, 6) = 7.108, *p* = 0.026], cis-aconitic acid [F(2, 6) = 7.369, *p* = 0.024], α-keto-glutaric acid [F(2, 6) = 15.627, *p* = 0.004], succinic acid [F(2, 3.966) = 27.45, *p* = 0.005], L-malic acid [F(2, 3.766) = 14.651, *p* = 0.017], and L-glutamine [F(2, 3.327) = 88.805, *p* = 0.001].

D-glucose-6-phosphate presented the highest electron flow activity (observed in the differentiated group) compared to all substrates (Figure 4b). The total electron flows for α-keto-glutaric, succinic acid, L-malic acid, and L-glutamine were higher (*p* < 0.05) in the differentiated than in the undifferentiated group. TRF treatment reduced the rate of electron flow for all substrates (*p* < 0.05).

## 3. Discussion

The RAW264.7 cells differentiated into osteoclast-like cells in the presence of PE particles. Although exposure to PE particles alone was able to induce differentiation [18], the addition of RANKL in this study was necessary to mimic the actual representation of the pathological environment in PE-induced osteolysis [54]. Nonetheless, a much more recent work, published by Song et al. (2019), has demonstrated that exogenous RANKL is essential for RAW264.7 cells to undergo osteoclastogenesis; no osteoclast formed in the absence of RANKL [55]. The size of PE particles used in this experiment was also within the required size for generating macrophage activity, which should be in the range of 0.3–10 µm [56]. In this study, a resorption assay was performed as a means of assessing the resorption activity of the differentiated osteoclast-like cells formed. It was found that a higher number of TRAP-positive cells was accompanied by more total resorption, consistent with the findings of our previous work [15].

The higher gene expression of RANK in osteoclasts exposed to PE particles observed in this study was consistent with other findings on the expression of RANK in the context of PE-induced osteoclastogenesis reported in the literature [12,57]. This receptor is essential for osteoclast differentiation, as the binding of RANKL to it initiates the signaling for osteoclastogenesis [58]. NF-κB and NFATc1 are two key transcription factors mediating osteoclastogenesis [6]. NFATc1 is known to mediate the expression of several osteoclast marker genes, such as *Itgb3*, *Calcr* [59,60], *Ctsk* [61], and Acp5 (TRAP-encoding gene) [62], as well as *Dcstamp* [63]. DC-STAMP is important for the multinucleation process during osteoclastogenesis [63]. The higher expression of some of these genes in the differentiated cells observed in the present study is consistent with findings previously reported in the literature [64]. The lower expression of those genes following TRF treatment demonstrated in this study may suggest the inhibition of osteoclast differentiation, and the activity seen earlier could be mediated through the down-regulation of the expression of those genes. This study is the first to demonstrate a reduction in PE-induced osteoclastogenesis following TRF treatment.

A higher level of ROS was observed in the differentiated cells. The lower ROS levels observed in the TRF-treated cells may suggest that treatment with TRF might have led to ROS clearance. The accumulated ROS could be generated by NADPH oxidases, which might have affected most cellular activities, including cell differentiation [65]. In this study, the NADPH oxidases that were highly expressed in cells exposed to PE and RANKL were *Nox1*, *Nox2,* and *Nox3*. The NADPH oxidase system functions as a critical player in intracellular ROS homeostasis [66]. There are seven members of the NOX family, including NOX1, NOX2, NOX3, NOX4, NOX5, DUOX1, and DUOX2. They bind NADPH and FAD, and are distinguished by specific catalytic subunits, interacting proteins, and sub-cellular localization [67]. The literature has indicated that NOXs are important in RANKL-mediated osteoclastogenesis [38].

*Nox1* is well-studied in terms of its effect in generating ROS. Sithole et al. (2021) have compiled evidence that a high level of *Nox1* contributed to osteoclastogenesis activity in RANKL-induced mice or cells [68]. The interaction of *Nox1* with nucleotide-binding oligomerization domain-2 (NOD2) leads to ROS generation in osteoclasts [69]. Inhibition of *Nox1* expression has been found to reduce RANKL-induced ROS production in BMM [35]. Rhaponticin—a compound found in the rhubarb rhizome—has been shown to inhibit osteoclastogenesis of RAW264.7 through suppression of *Nox1* expression and reduction of ROS activity. The suppression of NOX2 on RANKL-induced osteoclast differentiation was found to suppress ROS and superoxide anion production [70]. NOX2-derived superoxide enhanced RANKL-induced NFATc1 expression in osteoclasts [71]. The role of NOX3 in osteoclastogenesis or osteoclasts activity has not been studied in depth, thus far. A high expression of *Nox3* has been observed in RAW264.7 cells [72]. In this study, treating the differentiated cells with TRF reduced the expression of NOX1, NOX2, and NOX 3.

Nrf2 is a critical transcription factor that regulates the antioxidant enzymes in response to oxidative stress [66] and provides protection against mitochondrial toxins [40]. It was unexpected to see that the gene expression of NRF2 was higher in the differentiated cells than in the undifferentiated ones. The findings reported by Peng et al. (2018) suggested that exposure to PE particles did not necessarily result in down-regulation of antioxidant enzymes [39]. It has been shown that there was increased RANKL-induced osteoclast differentiation in Nrf2-deficient BMMs [73], which may suggest that the up-regulation of Nrf2 expression could decrease osteoclastogenesis. This is potentially supported by the finding in this study that the reduction in the number of TRAP-positive osteoclast-like cells following TRF treatment was accompanied by elevated Nrf2 expression. In mice liver, TRF treatment was shown to increase NRF2 gene expression in a dose-dependent manner [74]. Individual tocotrienol isomers have also been shown to increase *Nrf2* expression; for example, δ-tocotrienols reduced oxidative damage in an osteoblastic cell line through the PI3k/AKT-NRF2 pathway [75].

The accumulated ROS could also possibly be due to the increased activity of mitochondria in the group. ROS are produced during mitochondrial respiration, and mitochondria are one of the most significant ROS producers within the cell. A high level of ROS could, therefore, be correlated with the abundant number of mitochondria in the cells. It was observed, in this study, that differentiated cells had higher levels of both PCG-1α and PGC-1β compared to the undifferentiated cells. TRF lowered the mRNA expression of Pcg-1α but not PGC-1β. Even though PGC-1β has been studied in osteoclastogenesis more than PGC-1α, the finding of significant modulation of PGC1α in response to TRF treatment may suggest that PGC1α should not be overlooked. Both PCG-1α and PGC-1β are known to promote mitochondrial biogenesis [76,77]. PCG-1α has been studied more in osteoblasts [78,79,80], and has been shown to play a role in mitochondrial metabolism. Its over-expression has been found to affect the activity of Sirtuin 3 (a mitochondrial deacetylase), which controls the increase in osteoclast number by preventing osteoblast mitochondria from decreasing its function [78]. In an aging and neurodegenerative disease model, the high expression of PCG-1α led to the induction of mitochondrial metabolism and the removal of ROS by-products [81].

The high expression of PGC-1β in the differentiated group, compared to the undifferentiated group, together with changes in osteoclasts and mitochondrial activity could be supported by the few publications that have studied the involvement of PGC-1β in osteoclastogenesis. A previous study has shown that the gene expression of PGC-1β in wild-type murine BMMs was higher in RANKL-induced osteoclastogenesis [82]. Following the over-expression of iron-reducing hormone (Hepcidin), which led to suppression in osteoclast differentiation and bone resorption in BMM isolated from ovariectomized mice, it was found that the level of PGC-1β was low, accompanied with reduced levels of ROS and a lower number of mitochondria in cells [83]. A reduction in the level of PGC-1β and mitochondrial biogenesis were also observed during the suppression of RANKL-induced osteoclastogenesis and bone resorption following treatment with Cinchonine (CN), an antimalarial drug [84]. A previous study has demonstrated that osteoclastogenesis still occurred in PGC-1β-deficient mice; however, the osteoclasts showed impairment of resorption activity as a result of defects in the osteoclast cytoskeletal arrangement, and the cells were incapable of forming actin rings [85]. This is the first study to report the expression of PGC-1β during osteoclastogenesis following treatment with antioxidants.

The higher expression of *ATP5b* and *Cyc* in the differentiated group observed in the present study could reflect an increase in the formation of mitochondrial complexes during osteoclastogenesis. Apart from its structural role, *ATP5b* also regulates mitochondrial fission and fusion in mammalian cells [86]. Consistent with the abundance of mitochondria observed within osteoclasts, the higher expression of *ATP5b*, as seen in the differentiated cells here, is supported by findings from previous studies [26,82]. The structural function of Cytochrome C is to transfer electrons between complexes III and IV in the electron transport chain.

In this study, we demonstrated that there was higher ECAR in differentiated cells than in undifferentiated ones. This finding is in parallel with our later findings of higher D-glucose-6-phosphate oxidation and basal glycolysis observed in the differentiated group (refer to Figure 5). It has been suggested that it is typical to see an increase in ECAR followed by an increase in OCR [87], which was observed in the differentiated cells. Higher OCR and ECAR imply higher energy metabolism in the differentiated osteoclasts than the undifferentiated ones, which is in agreement with the previous literature [17,23]. Higher acidification usually refers to elevated lactate levels from glycolysis, even in aerobic conditions, and OXPHOS, which contributes to most energy production in osteoclast metabolism [28,87]. Reduction equivalents NADH and FADH2 produced from glycolysis might enter the ETC, thus increasing OXPHOS activity in the differentiated cells. The higher OXPHOS and acidification in the TRF-treated group might not be due to glycolysis, as the basal glycolysis was observed to be lower than in the differentiated group.

Most literature studying ETC considers the overall total OCR and ECAR; however, in this study, we further broke down events into several bioenergetic parameters [28]. Higher basal respiration was observed in differentiated cells, indicating higher ETC activity in these cells. This suggested that higher energy is needed in differentiated osteoclasts, compared to the undifferentiated osteoclasts. Proton leaks could lead to the generation of mitochondrial ROS [88]. A higher proton leak level in the differentiated group, together with increased ROS levels, was observed in our study.

The higher level of ROS in the differentiated group, as discussed earlier, could be associated with the high proton leak level observed in this study. The differentiated cells also showed low resistance upon exposure to FCCP, which could explain the low respiratory capacity observed in this group. Higher spare respiratory capacity leads to a more remarkable adaptation to stress [89]. During the spare respiratory capacity measurement, FCCP (an uncoupler) triggered a sudden proton rush across the inner mitochondrial membrane, disturbing the proton flux through FoF1-ATP synthase and increasing utilization of oxygen for maintenance of the H^+^ gradient [90]. The spare respiratory capacity could be influenced by other factors, such as mitochondrial membrane integrity, mitochondrial function, and biogenesis [91]. With TRF treatment, the maximal respiration was boosted, together with the increased spare respiratory capacity of the cells.

ATP is produced in glycolysis and ETC. Consequently, lactate and carbon dioxide (CO_2_) are produced, both of which contribute to acidification of the extracellular space. Our study demonstrated that the differentiated cells had higher glycolysis capacity and reserve than the undifferentiated and TRF-treated osteoclasts. This may suggest that glucose utilization, as the main energy substrate, was high, in parallel with the high metabolism expected in the differentiated cells [92]. Li et al. (2020) [28] have reported that both progenitors and mature osteoclasts of BMM favored the utilization of glucose for energy, instead of glutamine or fatty acids. They also found that GLUT1 was highly expressed in osteoclasts and that lactate was produced even during aerobic respiration. The increase in glycolysis capacity and reserve was supported by our data regarding the higher oxidation profile of D-glucose-6-phosphate in the differentiated group. Kim et al. (2007) [23] have shown that osteoclast precursors gained high glucose metabolism at an early stage of RANKL-stimulated osteoclast differentiation, suggesting the strong role of glycolysis in differentiation.

Examination of the MitoPlate, comprised of a panel of substrates from various metabolic pathways such as glycolysis, the TCA cycle, ETC, malate aspartate shunt (MAS), and β-oxidation [93], allowed us to determine that substrates such as D-glucose-6-phosphate, cis-aconitic acid, α-keto-glutaric-acid, succinic acid, L-malic acid, and L-glutamine had different oxidation profiles between groups. These substrates contribute to the production of reducing equivalents (NADH and FADH2, which donate electrons to complex I and II of the ETC, respectively) and ATP by entering glycolysis, TCA, or by direct oxidation of the substrate. D-glucose-6-phosphate is a main substrate in glycolysis, which is the main metabolic pathway for both pre-osteoclasts and differentiated osteoclasts [17,23,28,92]. However, the cells could also shift to OXPHOS, in which other substrates, such as galactose [17] and lactate from the glycolytic pathway [29], are consumed. Meanwhile α-ketoglutarate, succinate, cis-aconitic acid, and L-malate are intermediate molecules of the TCA pathway, which is an important pathway for ATP production in the mitochondrial matrix for osteoclasts [17,27]. Evidence of the involvement of L-glutamine in osteoclastogenesis has been put forward by Indo et al. (2013); in their study, glutamine transporter (Slc1a5) was found to have high expression when early osteoclasts transitioned from BMM [27]. A later study found that the glutaminase enzyme—which converts glutamine to glutamate and, subsequently, to α-ketoglutarate—had high expression during osteoclastogenesis. They also found that the addition of a membrane-permeable α-ketoglutarate analogue restored osteoclastogenesis under glutamine-deficient conditions [94]. It has been reported that supplementation of α-ketoglutarate was also able to reduce the serum levels of type 1 collagen cross-linked C-telopeptide (CTX-1) and increase osteocalcin in ovariectomized rats, protecting the animals from osteopenia and osteoporosis [95]. Higher L-malic acid oxidation in the differentiated group was expected, as a previous study has reported that the enzyme converting L-malic acid into oxaloacetate (a substrate of TCA cycle) was highly expressed when cells were induced by RANKL [96]. The conversion of succinate into fumarate produces FADH2 [97,98]. Guo et al. (2017) [99] have shown that succinate treatment induces osteoclastogenesis in RAW264.7 through NF-κB and restored osteoclast formation in BMM of metformin-treated mice; however, the direct effect of the compound on bone metabolism was not studied. The higher succinate oxidation seen in this study could indicate that there was an increased function of complex II in the differentiated group. This study is the first to report data on cis-aconitic acid as a substrate in osteoclasts.

## 4. Materials and Methods

### 4.1. Preparation of PE Particle

The PE particles that were used in this experiment (Ceridust 3620, Clariant Pte. Ltd., Louiseville, KY, USA) were subjected to particle size analysis using a Zetasizer (Malvern Instruments Ltd., Malvern, UK). Six-well plates were used. Before coating the well plates, PE particles were washed with 95% ethanol and air-dried in an oven. PE particles resuspended in ethanol were coated onto the well using the method described by Sartori et al. (2017) [100], and two milliliters of reconstituted PE in ethanol (0.5 mg/mL) was pipetted into each corresponding well. The plates were then left to dry overnight in the incubator. On the day before seeding the cells, the plates were exposed to UV for an hour. Plates were then washed with HBSS twice, in order to remove any ethanol remaining in the wells.

### 4.2. RAW264.7 Culture

Murine monocyte/macrophage cells (RAW264.7) were purchased from the American Type Culture Collection (ATCC; Manassas, VA, USA). In the corresponding wells, cells were grown in the PE-coated wells. Cells were seeded at a density of 2.6 × 10^4^ cells/cm^2^, consistent with Sartori et al. (2017). Cells were maintained in DMEM (Hyclone, Logan, UT, USA) supplemented with 10% fetal bovine serum (Gibco, Waltham, MA, USA), pyruvate, 1% penicillin/streptomycin (Gibco, Waltham, MA, USA), and 2 mM L-Glutamine (Gibco, Waltham, MA, USA). Due to unavoidable technical reasons, cells at passage number 17 were used for the experiments; this high passage number of cells used is acknowledged as a limitation of the study, even though there is literature indicating that RAW264.7 cells with passage number of 20 and below are still capable of differentiating into osteoclasts [101]. To allow for differentiation of the cells, the medium was switched from high-glucose DMEM to α-MEM (Gibco, Waltham, MA, USA). RANKL (at 100 ng/mL) was added to the culture media, starting from day 0, in order to induce differentiation. Undifferentiated cells were allowed to grow up to day 9 in the same medium in the absence of RANKL. The third group, named the TRF-treated group (see below), was included in the study for comparison with the differentiated and undifferentiated groups. Cells were maintained in an 8% CO_2_ incubator at 37 °C.

### 4.3. Preparation of TRF

The Gold Tri E 70 used in this study was purchased from Sime Darby Bioganic Sdn. Bhd., Kuala Lumpur, Malaysia. The TRF was comprised of about 75% tocotrienols and 25% α-tocopherol. The tocotrienols-rich fraction compound was prepared based on the method described by Jaafar et al. (2018) [42]. In this study, 50 µg/mL of TRF was the dose used, which was determined as the optimal dose in our (unpublished) preliminary work.

### 4.4. Gene Expression Study

The primers used in this study were designed using the Primer-Blast software from NCBI. Before RNA extraction, cells were washed with PBS twice. The extraction of total RNA was performed using TRI Reagent^®^ (Sigma-Aldrich, St. Louise, MI, USA). The sample was checked for purity using a Nanodrop 2000c spectrophotometer. Complementary DNA (cDNA) synthesis was carried out in a thermal cycler (SelectCycler II, New York, NY, USA) using a Qiagen™ Quantinova^®^ (QIAGEN, Hilden, Germany) reverse-transcription kit. The procedure was performed according to the manufacturer’s protocol.

The sequences of the polymerase chain reaction (PCR) primers used in this study are listed in Table 1. The cDNA were amplified in a qPCR (Biorad CFX384™ Real-Time System, Hercules, FL, USA) machine using a Qiagen Quantinova™ SYBR^®^ Green PCR kit (QIAGEN, Hilden, Germany). cDNA template, at a concentration of 100 ng, and primer, at a concentration of 0.7 µM, was used for the amplification in a total reaction volume of 10 µL. A two-step cycling PCR method performed with denaturation was set at 95 °C for 5 s, and annealing/extension was set at 60 °C for 10 s. This was repeated for 40 cycles. All primers were normalized against the glyceraldehyde-3-phosphate dehydrogenase (GAPDH) gene.

### 4.5. Tartrate-Resistant Acid Phosphatase (TRAP) Staining

Cells were stained using a TRAP staining kit purchased from Cosmobio (Tokyo, Japan). Cell culture medium was removed, and cells were washed once with PBS. Then, 100 µL of 10% neutral buffer formalin was added to the wells, and cells were fixed for 5 min at room temperature. Staining of cells was performed following the procedure recommended by the protocol. The plate was then viewed using an EVOS FLc (Thermo Fisher Scientific, Waltham, MA, USA) microscope and an Olympus BX53 (Olympus, Shinjuku City, Japan) microscope with a polarizing lens.

### 4.6. Bone Resorption Assay

The bone resorption assay kit (Cat. No. CSR-BRA-48KIT) was purchased from Cosmobio (Tokyo, Japan). The kit contains 48-well plates pre-coated with carbonate apatite (CaP). Prior to cell seeding, each well in the plate was coated with fluoresceinamine-labelled chondroitin sulphate (FACS) for 2 h. RAW264.7 cells were seeded based on the recommended density of the kit. Incubation and assay procedures were performed as recommended by the manufacturer. An EnSpire^®^ multimode plate reader (PerkinElmer, Waltham, MA, USA) was used to read the fluorescence signal produced. Wavelengths were set at 485 nm for emission and 535 nm for excitation.

### 4.7. MitoPlate Assay

A total of 30 µL of the assay mix was pipetted into each well. The plate was then incubated at 37 °C for 1 h, in order to dissolve the substrates pre-coated on the plate. Cells were prepared and suspended in 1× Biolog MAS (Biolog Inc., Hayward, CA, USA) media at a concentration of 3 × 10^6^ cells/mL, and 30 µL of cell suspension was added to each well. Then, 75 µg/mL Saponin was added into each well, in order to permeabilize cell membranes. The plate was then loaded onto an EnSpire^®^ multimode plate reader (PerkinElmer, Waltham, MA, USA) and kinetic measurements were taken every 5 min for 6 h at a wavelength of 590 nm. All measurements were normalized against the control wells not containing any substrate.

### 4.8. Mitochondrial Stress Test

Using a Seahorse XFe96 Extracellular Flux Analyzer (Agilent Technologies, Santa Clara, CA, USA), we measured the oxygen consumption rate and extracellular acidification rate of cells. The day before the experiment, 7 × 10^4^ cells in 80 µL suspension were seeded into each well (except for the background well) and incubated for 24 h in a standard CO_2_ incubator at 37 °C. The medium in each well was changed to Agilent Seahorse XF DMEM (pH 7.4) supplemented with 1mM pyruvate, 2 mM glutamine, 10 mM glucose, and all necessary compounds needed, based on the group.

The running protocol was set as three readings for baseline and after each event, with measurements of the oxygen consumption rate (OCR) and extracellular acidification (ECAR) made for two minutes and at three-minute intervals. Three events were included: the first event was the injection of 1 µM of oligomycin, the second event was the injection of 1 µM Carbonyl cyanide-4- (trifluoromethoxy) phenylhydrazine (FCCP), and the third event was the injection of 0.5 µM rotenone/antimycin. These inhibitors were purchased from Agilent Technologies (Santa Clara, CA, USA). The oxygen consumption rate (OCR) and extracellular acidification rate (ECAR) were calculated using the WAVE software version 2.4.0.60 (Agilent Technologies, Santa Clara, CA, USA).

### 4.9. Glycolysis Stress Test

The cells were seeded (7 × 10^4^ cells per well) on the day before the assay started. On the following day, the medium in each well was replaced with the assay medium, consisting of Agilent Seahorse XF DMEM (pH 7.4) added with 2 mM L-glutamine without the addition of pyruvate and glucose. The first event started with the injection of 10 mM glucose from Port A, followed by the injection of 1 µM oligomycin, while the final event was injection of 10 mM 2-deoxyglucose (2-DG). All Seahorse XF Media, supplements, and calibrant were purchased from Agilent Technologies (Santa Clara, CA, USA). Measurements of baseline and after-events were conducted as stated in Section 4.8.

### 4.10. Statistical Analysis

All the data were subjected to a Shapiro–Wilk normality test before hypothesis testing. ANOVA followed by Tukey’s multiple comparisons test was performed when the data were normally distributed, while non-normally distributed data were subjected to Welch ANOVA followed by a Games–Howell multiple comparisons test. Parametric *t*-tests were performed for the comparisons of two groups. Confidence intervals were set at 95%, and a *p*-value less than 0.05 was considered to indicate statistical significance.

## 5. Conclusions

From this study, it was found that there were higher levels of ROS and changes in the expression of oxidative stress-associated genes during PE-induced osteoclast formation and activity. These changes were accompanied by changes in the metabolism of the cells, including mitochondrial respiration, glycolysis, and substrate oxidation. Treatment with TRF modulated some of these changes.

## Figures and Tables

**Table 1:** Sequences of the primers used in the gene expression study.

Gene	Sense (5′→3′)	Antisense (5′→3′)
*Traf6*	AAA GCG AGA GAT TCT TTC CCT G	ACTGGGGACAATTCACTAGAGC
*Rank*	GCC CAG TCT CAT CGT TCT GC	GCA AGC ATC ATT GAC CCA ATT C
*Ctsk*	TGG AGT TGA CTT CCG CAA TCC	CCC ACA TCC TGC TGT TGA GAA T
*Atp5b*	AGT TGC TGA GGT CTT CAC GG	GGA GAT GGT CAT ATT CAC CTG C
*Nox 1*	AAG CCA TCC TCA CAA TTG TTC C	AGG ATC CAC TTC CAA GAC TCA G
*Nox 2*	TTC CAG TGC GTG TTG CTC G	GTG CAA TTG TGT GGA TGG CG
*Nox 3*	AACAAGTGTGTGCTGTAGAGG	TCCAGGTTGAACAAGTGTGCC
*Nox 4*	TAC TTC CAA GAT GAA CCA TGC C	GGA ATC GTT CTG TCC AGT CTC C
*Nfatc1*	ACA TGC GAG CCA TCA TCG AC	TGT GAA CTC GGA AGA CCA GC
*Gapdh*	AGGTCGGTGTGAACGGATTTG	TGTAGACCATGTAGTTGAGGT
*Pgc1a*	CTTGTGTCAAGGTGGGC	TGAGGTGCTTATCGAGTTCCG
*Pgc1b*	CTTGTGTCAAGGTGGATGGC	TGAGGTGCTTATGCAGTTCCG
*Cyc*	GAA CAA GTG TGG TTG CAC CG	AGCTTCGGACTCGAAGACAG
*Nrf2*	AGT GGATCCGCCAGCTACTC	GCAAGCGACTCATGGTCATC
*Calcr*	CACTGCTAAGGAGAGCCAGC	TGAGGCGCAGAAGTAAGCAC
*Itgb3*	GTGGAAGAGCCTGAGTGTCC	AGATGAGCAGAGTAGCAAGGC
*Dcstamp*	ACGTGGAGAGCAAGGAACC	TCTCAGACACACTGAGACGTG

## Data Availability

The data presented in this study are available on request from the corresponding author. The data are not publicly available as findings from an ongoing work, which is a continuation of the work presented here, have not yet been published.
